# Single phase charge ordered stoichiometric CaFe_3_O_5_ with commensurate and incommensurate trimeron ordering

**DOI:** 10.1038/s41467-019-13450-5

**Published:** 2019-12-02

**Authors:** Simon J. Cassidy, Fabio Orlandi, Pascal Manuel, Simon J. Clarke

**Affiliations:** 1 Department of Chemistry, University of Oxford, Inorganic Chemistry Laboratory, South Parks Road, Oxford, OX1 3QR UK; 2 ISIS Facility, STFC Rutherford Appleton Laboratory, Harwell Oxford, Didcot, OX11 0QX UK

**Keywords:** Inorganic chemistry, Materials chemistry, Electronic properties and materials, Phase transitions and critical phenomena

## Abstract

Mixed-valent transition metal compounds display complex structural, electronic and magnetic properties which can often be exquisitely tuned. Here the charge-ordered state of stoichiometric CaFe_3_O_5_ is probed using neutron powder diffraction, Monte Carlo simulation and symmetry analysis. Magnetic ordering is dominated by the formation of ferromagnetic Fe^3+^–Fe^2+^–Fe^3+^ trimers which are evident above the magnetic ordering transition. Between *T*_N _= 289 K and 281 K an incommensurate magnetically ordered phase develops due to magnetic frustration, but a spin Jahn-Teller distortion lifts the frustration and enables the magnetic ordering to lock in to a charge-ordered commensurate state at lower temperatures. Stoichiometric CaFe_3_O_5_ exhibits single phase behaviour throughout and avoids the phase separation into two distinct crystallographic phases with different magnetic structures and Fe valence distributions reported recently, which likely occurs due to partial Fe^2+^ for Ca^2+^ substitution. This underlines the sensitivity of the magnetism and chemistry of these mixed-valent systems to composition.

## Abstract (web summary)

A significant challenge in understanding the behaviour of strongly correlated materials is their sensitivity to composition. Here the authors show that stoichiometric CaFe_3_O_5_ avoids the electronic phase separation reported in a non-stoichiometric sample, and also exhibits an incommensurate magnetic phase.

## Introduction

Mixed-valent compounds of iron and other transition metals show a wide range of physical and chemical properties. Magnetite, Fe_3_O_4_, has long been known to undergo the Verwey transition associated with localisation of Fe^2+^ and Fe^3+^ states^1^. Modern instrumentation was able to detect anomalous shortening of some Fe–Fe distances at this transition, consistent with localisation of the minority Fe^2+^ spin into linear three-Fe clusters named trimerons^2^, thus resolving the complex nature of this transition^3^. CaFe_3_O_5_ is another mixed-valence compound of iron with a mean Fe oxidation state of +2.66, the *n* = 1 member of a series of general formula CaFe_2_O_4_(FeO)_*n*_. These compounds consist of CaTi_2_O_4_-type CaFe_2_O_4_ slabs, separated by rock salt-type FeO slabs of a thickness that increases with increasing *n*. *n* is known to take integer values of 1, 2 and 3^4^, and half-integer mean values of 1.5 and 2.5 are found when there are alternating (FeO)_*n*_ and (FeO)_*n*+1_ blocks^5^. The *n* *=* 3 member of the series, CaFe_5_O_7_, exhibits a complex structure^6^, stacking faults that are synthesis dependent^7,8^, complex long range magnetic order and Charge Order (CO), an important phenomenon in mixed-valent compounds^9^. Charge ordering has also been observed in two compounds that are isostructural with CaFe_3_O_5_: Fe_4_O_5_^10^, a high-pressure-synthesised mixed-valence compound in which charge ordering occurs in Fe dimers and trimers, and MnFe_3_O_5_, which undergoes charge ordering with localisation of the charges on distinct Fe^2+^ and Fe^3+^ sites^11^.

**Fig. 1:**
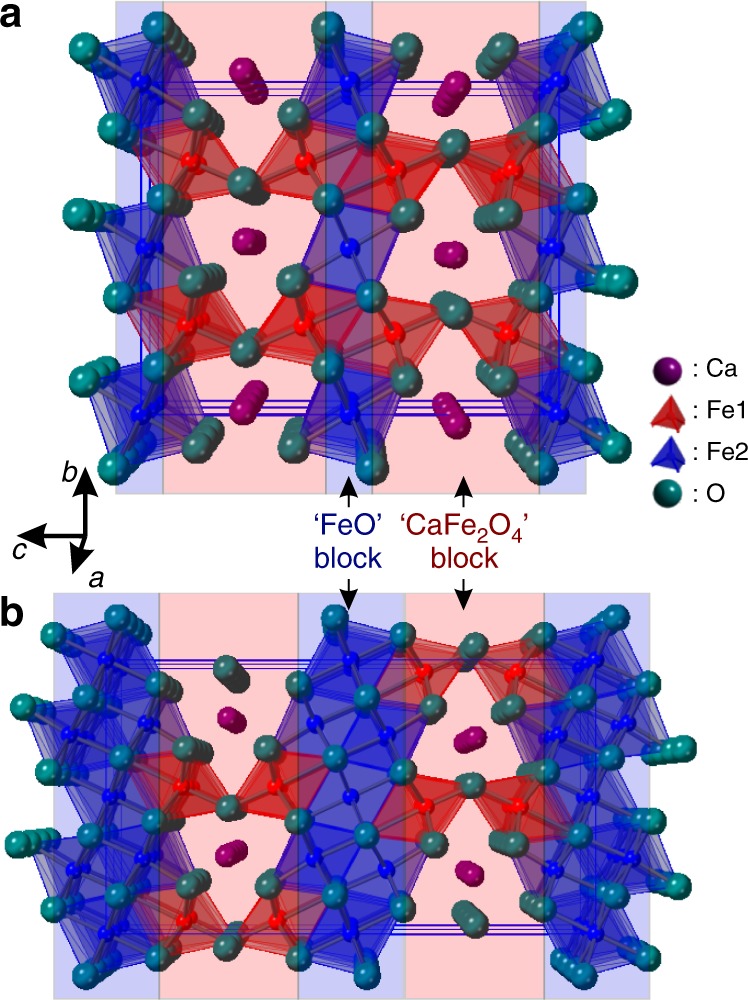
Nuclear structure of CaFe_2_O_4_(FeO)_*n*_ compounds. **a** structure of CaFe_3_O_5_ (*n* = 1) in *Cmcm* symmetry and **b** structure of CaFe_5_O_7_ (*n* = 3) (at > 360 K) in *Cmcm* symmetry. CaFe_3_O_5_ has two Fe sites: Fe1 on the 8*f* Wyckoff site and Fe2 on the 4*a* Wyckoff site. The Fe1 site can be thought of as being in a CaFe_2_O_4_ block, while the Fe2 site is in an FeO block, and these blocks stack along the *c* axis.

CaFe_3_O_5_ adopts an orthorhombic structure in space group *Cmcm* with two crystallographically distinct iron sites. Fe1 is an octahedral site (8*f*) in the CaFe_2_O_4_ layer and Fe2 is an octahedral site (4*a*) in the FeO layer. These layers stack along the *c* axis as shown in Fig. 1a. Gerardin et al. used Mössbauer spectroscopy to characterise antiferromagnetic order below a *T*_N_ of 282 K^12^, and suggested a Charge Averaged (CA) state pertaining above *T*_N_ and a CO state below, with localisation of Fe^3+^ and Fe^2+^ on distinct sites. More recent work by Hong et al. confirms the high temperature CA state, but they report a macroscopic phase separation in their sample below the magnetic ordering transition (reported as *T*_M_ = 302 K)^13^, They used high resolution diffraction data to clearly observe a single phase above this *T*_M_ which separates into two phases below. Bond valence sums for the Fe sites suggested that the minority phase remains charge averaged (CA) below the magnetic ordering transition, while the majority phase is charge ordered (CO) with the Fe1 site characterised as Fe^3+^ and the Fe2 site as Fe^2+^, reflecting the expectation of the work of Gerardin et al.^12^. The CA and CO phases both exhibit long range magnetic ordering, clearly presenting separate sets of magnetic Bragg peaks in the powder neutron diffractograms. Hong et al.^13^. show in their analysis of bond lengths that the CO phase meets the requirements for trimeron formation by means of localising the minority spin in Fe1-Fe2-Fe1 units^2,13^.

The evidence for the phase separation in the work by Hong et al. is unquestionable and well characterised but its origin is unclear^13^, and it is unusual that it was not detected in the Mössbauer work by Gerardin et al.^12^. From the Rietveld refinements the sample of Hong et al.^13^. contains 4% substitution of excess Fe for Ca (making the overall composition from the refinements Ca_0.952(8)_Fe_3.040(8)_O_5_), which they suggest may tip the balance between the energies of the CO and CA states, raising the possibility that the occurrence of the phase separation may be sample dependent, and may be sharply dependent on composition. A further recent study of CaFe_3_O_5_ as a potential oxide-ion storage material in solid oxide fuel cells also used Mössbauer spectroscopy and was largely consistent with the work of Gerardin et al.^12^, detecting only one phase at low temperature^14^. However, it also noted that the room temperature Mössbauer spectrum deviated from the usual 3:2:1 ratio for magnetic features, and concluded that an incommensurate antiferromagnetic hyperfine field distribution was the simplest model to account for the spectrum.

Here we report that stoichiometric samples of CaFe_3_O_5_ (see Methods section and Supplementary Table 1 for synthetic details) lack the long range phase separation reported by Hong et al.^13^ and contain solely the trimeron-CO phase below the magnetic ordering transition. The analysis of the degree of charge-separation between the crystallographic Fe sites from their ordered moments results in a model consistent with the model for charge-ordering by localisation of the minority spin on distinct Fe^2+^ sites from the Mössbauer measurements of Gerardin et al.^12^ and the localisation of the minority spin within the trimeron units proposed by Hong et al. from Neutron and X-ray diffraction^13^. We find that the onset of long range magnetic ordering in this phase proceeds via an incommensurately ordered region, in which the trimerons persist in a modulated form. We account for the origin of this incommensurate state and its “lock-in” to the commensurate long range magnetically ordered state on further cooling and formally derive the symmetry of the magnetically ordered state. Furthermore Monte-Carlo simulations using realistic sets of exchange interactions confirm the symmetry requirement of a spin Jahn-Teller distortion to allow the adoption of the experimentally observed commensurate ground state. These simulations also suggest that the trimeron formation starts well above *T*_N_, as shown by the magnetic diffuse scattering and by the evolution of the cell parameters, and is the driving force for adoption of the observed magnetic structure in stoichiometric charge ordered CaFe_3_O_5_.

## Results

### Crystal structure analysis

**Fig. 2:**
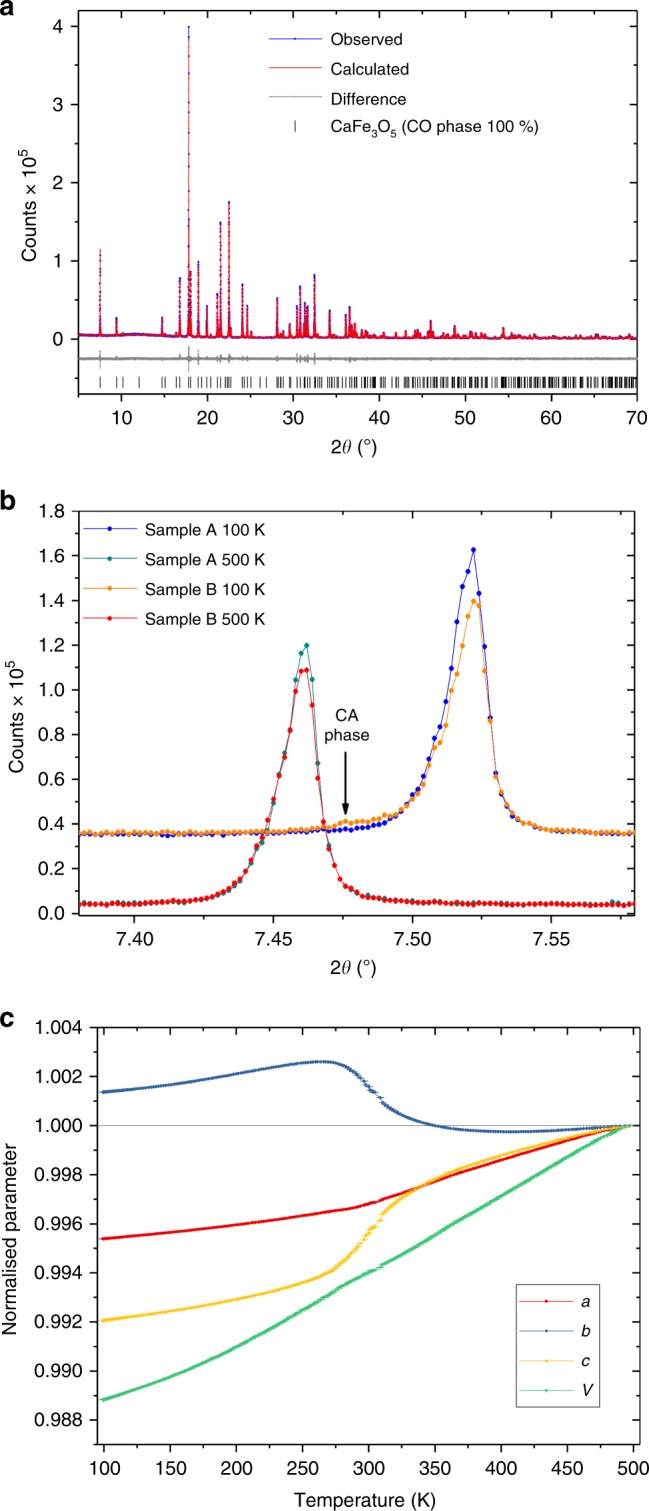
Synchrotron X-ray diffraction data. **a** Rietveld fit to high resolution synchrotron powder X-ray diffraction data (sample A) at 100 K using a single phase of CaFe_3_O_5_ in space group *Cmcm*. **b** (002) peaks of CaFe_3_O_5_ samples at 500 K and 100 K (offset in the *y*-axis by 30000 counts) showing no visible phase separation between these temperatures for sample A and a very slight degree of phase separation in the 100 K data for sample B, corresponding to the CA phase reported by Hong et al.^13^, which occurs at a much higher phase percentage (30–50%) in their sample. **c** Temperature dependence of the cell parameters across the Charge Ordering transition in sample A. Parameters were obtained by Rietveld refinement against diffraction patterns obtained on the I11 diffractometer (Diamond Light Source)^22^, collected continuously with the PSD detector while cooling the sample with a cryostream between 500 and 100 K.

Powder X-ray diffraction (PXRD) patterns of multiple samples of CaFe_3_O_5_ (see Methods section and Supplementary Table 1), were measured at 100 and 500 K using the high-resolution MAC detector of I11 at the Diamond Light Source (Fig. 2). We found evidence in some samples for the phase separation into CO and CA phases described by Hong et al.^13^ but of five samples measured, the maximum amount of the CA phase at 100 K was estimated to be 2.7% (in sample B); much lower than the ~40% reported in the sample of Hong et al.^13^. In three samples, including the sample measured by neutron diffraction (sample A) there was no detectable CA phase in the PXRD, showing that the synthesis route reported here (which differs from that of Hong et al.^13^) reliably produces samples which yield only the CO phase at low temperatures.

Aside from the phase separation evident in sample B, no peak splitting or superstructure reflections were observed as a function of temperature and Rietveld refinements were consistent with the structure maintaining *Cmcm* symmetry above and below the magnetic transition.

The compound undergoes anisotropic thermal contraction on cooling associated with long range magnetic ordering (Fig. 2c), which is also observed in isostructural Fe_4_O_5_ and MnFe_3_O_5_ at their respective magnetic ordering temperatures^10,15^. In CaFe_3_O_5_ the lattice parameters begin to deviate from linearity on cooling at around 410 K, well above the long range magnetic ordering temperature established by neutron diffraction, indicating that short range magnetic correlations influence the crystal structure well above the onset of long range order. From ~410 K down to 280 K the *b* lattice parameter (10.01770(3) Å at 500 K) undergoes an expansion, whilst the *c* lattice parameter (12.67159(3) Å at 500 K) undergoes a complementary contraction, conserving the unit cell volume. The *a* lattice parameter (3.039760(7) Å at 500 K) and the unit cell volume change comparatively smoothly with only slight inflections at the transition. Each of the lattice parameters then contracts smoothly as a function of temperature below 280 K, after long-range magnetic order is established. This behaviour of the lattice parameters quantitatively matches the reported behaviour for the CO phase reported by Hong et al.^13^.

### Magnetometry

**Fig. 3:**
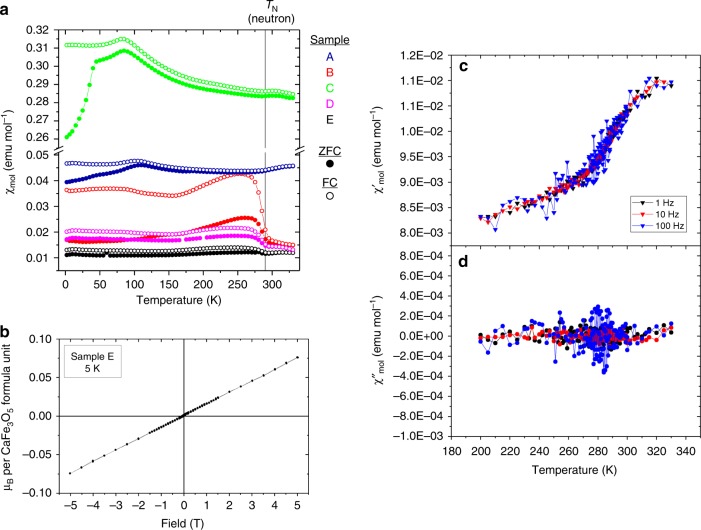
Magnetometry measurements. **a** Susceptibility versus temperature for five samples of CaFe_3_O_5_ measured in 1000 Oe fields. The transition at the marked *T*_N_ is a property of the main phase, while the absolute values of the susceptibility and transitions at lower temperatures in some samples appear to be strongly influenced by minuscule quantities of more strongly magnetic impurities such as Fe_3_O_4_ and Ca_2_Fe_2_O_5_ **b** Magnetisation Isotherm of sample E at 5 K, showing a low moment and no hysteresis, consistent with antiferromagnetic behaviour. **c** and **d** real and imaginary parts of the AC susceptibility of sample A which show no ferromagnetic component to the transition at *T*_N_.

All samples of CaFe_3_O_5_ show magnetic transitions between 285 and 310 K in the susceptibility data (see Methods section). The behaviour varies from sample to sample (Fig. 3); some samples show purely a small downturn in the susceptibility with decreasing temperature, suggestive of an antiferromagnet, with a maximum in the first derivative of the susceptibility with temperature at 300 K. Other samples show a much larger upturn in the susceptibility on cooling, typical of a weakly ferromagnetic component, with the maximum in the value of the first derivative of the susceptibility at 285 K. This variation correlates with the degree of phase separation in the sample. The conclusion is that the CO phase is purely antiferromagnetic, consistent with the magnetic symmetry arguments below, while the minority CA phase has a ferromagnetic component. Our Sample B contains ~3% of the CA phase according to the diffraction data and exhibits a rise in the field-cooled susceptibility of 0.02 emu mol^−1^. The sample of Hong et al. measured under similar conditions exhibited a ~0.2 emu mol^−1^ rise in susceptibility for a ~40% fraction of the CA phase^13^, confirming a quantitative correlation between CA phase percentage and the size of the ferromagnetic feature in the susceptibility. This suggests that the presence of the CA phase can be reliably quantified using magnetometry in smaller amounts than is possible using X-ray diffraction. The sample probed using neutron diffraction (sample A) shows no rise in the DC susceptibility at 295 K on cooling and measurements carried out in a low (3.5 Oe) AC field show a very weak, broad feature in the real component that appears to be frequency independent and has no imaginary signal, confirming the lack of a ferromagnetic component. Sample A’s magnetisation isotherm is complicated by a <0.5% Fe_3_O_4_ impurity, but sample E, which contains no Fe_3_O_4_ and an estimated 0.2 % of the CA phase shows linear magnetisation versus field curves at both 300 K and 5 K, with no hysteresis when sweeping the field between −5 and +5 T, consistent with the CO state being antiferromagnetic at 5 K.

### Long range magnetic ordering

Sample **A** showed strong magnetic Bragg peaks in the neutron powder diffractogram at 5.5 K with no diffuse features, consistent with long-range magnetic order. These peaks index to a single propagation vector of (½, 0, 0) putting the **k** vector along the Σ(α00) symmetry line.

**Fig. 4:**
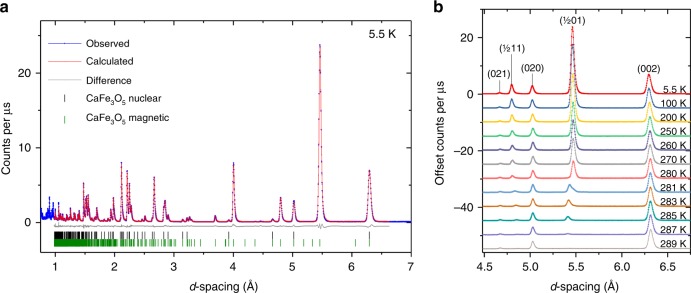
Neutron powder diffraction measurements of CaFe_3_O_5_. **a** Rietveld refinement of sample A at 5.5 K shown against the 90° detector bank of the WISH neutron diffractometer^19^. **b** Evolution of the magnetic reflections with temperature.

ISODISTORT was used to explore the possible magnetic space groups consistent with the parent symmetry and the observed propagation vector^16^, and TOPAS Academic was used to perform Rietveld refinements against the neutron powder diffraction (NPD) data, testing each of the symmetry adapted modes as a refined parameter^17,18^. The magnetic space group *P*_*a*_*bca* (61.438) (BNS notation)^19^, corresponding to the order parameter mΣ_3_(ξ, − ξ) produced excellent agreement with the data, describing ordered moments on both Fe sites with a single value of the moment on each Fe sub-lattice. The other possible space groups *P*_*c*_*bcm* (57.388) and *P*_*c*_*ca*2_1_ (29.106) gave comparable fits but do not give the single value of the moment on each Fe sub-lattice expected for an oxide with localized moments. The *P*_*c*_*bcm* (57.388) model allows an ordered moment on only half of the Fe ions, inconsistent with previous Mössbauer results^12^, while the *P*_*c*_*ca*2_1_ (29.106) model has four independent Fe sites with strongly correlated values of the moments making the refinement very unstable. We discuss this point further below. Our magnetic structure in *P*_*a*_*bca* was derived independently of the work of Hong et al. and is consistent with their CO model^13^. Using the data from WISH with sample A, which shows single phase behaviour in the magnetically ordered regime, we refined stably separate magnetic moments for the two independent Fe sites as discussed below. The Rietveld refinement, shown in Fig. 4a, gives good agreement with a single nuclear phase and the CO magnetic phase. The CA magnetic phase reported by Hong et al.^13^ has a **k** = (0, 0, 0) propagation vector and would add intensity to nuclear reflections, particularly the (0 2 1) peak, but in our sample this peak is weak and temperature independent showing that it results purely from a nuclear contribution to the Bragg scattering in sample A (Fig. 4b).

**Fig. 5:**
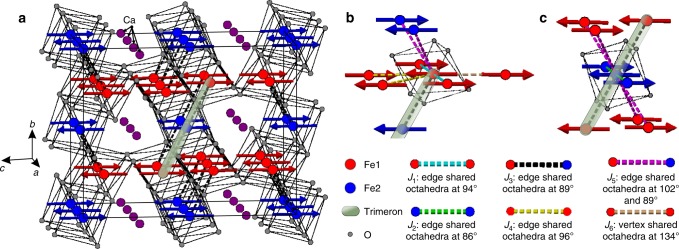
Magnetic ground state and main exchange interactions of charge ordered CaFe_3_O_5_. **a** Magnetic structure of charge-ordered CaFe_3_O_5_ between 280 and 5 K with the magnetic unit cell denoted by the black box and the trimerons shown with black dashed lines (which also denote the *J*_3_ interactions) between Fe centres, with one of them further highlighted by the shaded ellipse. **b** Coupling interactions experienced by Fe1 to other Fe sites. **c** Coupling interactions experienced by Fe2 to other Fe sites. The type of coupling interaction is denoted by the colour of the bond as shown in the key.

The magnetic structure is shown in Fig. 5, and can be described as an antiferromagnetic arrangement of the trimeron units along the cell axes. These (Fe1)^3+^-(Fe2)^2+^-(Fe1)^3+^ linear units (highlighted in Fig. 5a) are formed via a ferromagnetic superexchange interaction (*J*_3_) generated through edge-shared octahedra with 89° Fe1-O-Fe2 linkages. The antiferromagnetic coupling of these trimeron units along the *a*-axis is through the *J*_1_ and *J*_2_ exchange paths (94° Fe1-O-Fe1 and 86° Fe2-O-Fe2 linkages between edge-sharing octahedra respectively), whereas the coupling along the *c* direction (*J*_6_) is between (Fe1)O_6_ octahedra through vertex-shared linkages with a 134° Fe1-O-Fe1 angle. Another two pairs of couplings, *J*_4_ and *J*_5_, between edge-sharing octahedra become inherently frustrated as a result of the ordering. The pair of *J*_4_ interactions couples Fe1 to two further Fe1 through shared edges with identical Fe1-O-Fe1 linkages, but one of the pair of interactions is ferromagnetic and the other antiferromagnetic, which is inevitable since the coupled sites are offset from each other by ½ along *a*. Similarly Fe1 is linked to two Fe2 by the pair of *J*_5_ interactions with one being ferromagnetic and the other antiferromagnetic. The two components of each of these frustrated exchange paths (yellow and purple bonds in Fig. 5b, c), are equivalent in *Cmcm* since they are related by the mirror plane perpendicular to the *a*-axis. In order to develop long range order the system must resolve the frustrations, and this is possible through a so-called spin Jahn-Teller distortion^20^, The magnetic order parameter mΣ_3_(ξ, − ξ) can couple a displacive distortion with propagation vector *q* *=* (100) to conserve the lattice translational symmetry. This distortion will transform as the Y_3_^−^(σ) irreducible representation of the parent space group and will reduce the nuclear symmetry to *Pbcm*, effectively breaking the mirror plane perpendicular to *a*, and making each component of the two pairs of frustrated interactions, *J*_4_ and *J*_5_, inequivalent. The coupling between the two order parameters in the free energy can be written as −σξ^2^, indicating that the displacive distortion is a secondary order parameter induced by the magnetic ordering and its amplitude will be very small, explaining why it is not evident in the diffraction data.

**Fig. 6:**
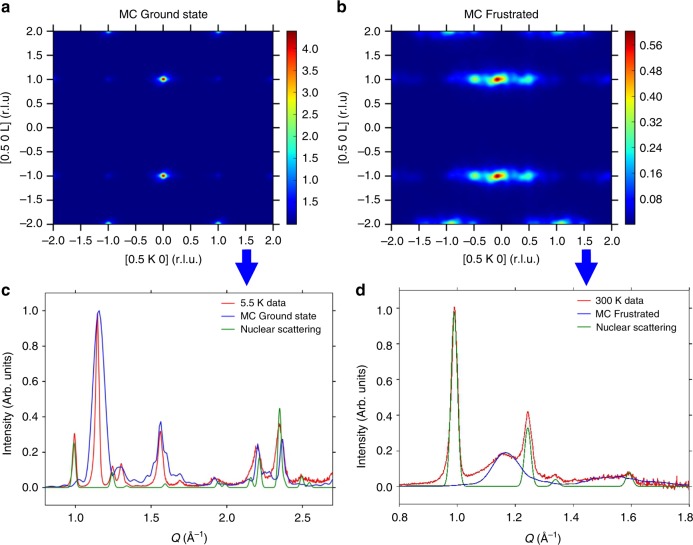
Magnetic scattering calculated from the MC simulation for charge ordered CaFe_3_O_5_. The upper panels show the 0.5*KL* scattering plane calculated from the MC simulations with (i.e. “MC Ground State”) (**a**) or without (i.e. “MC Frustrated”) (**b**) the Y_3_^−^(σ) distortion that relieves the frustration of the *J*_4_ and *J*_5_ exchange paths (See also Supplementary Fig. 1). The lower panels show experimental neutron powder diffractograms (red) of sample A alongside the corresponding Monte Carlo computation of the powder average nuclear (green) and magnetic (blue) scattering. The simulation with the Y_3_^−^(σ) distortion (**a**) shows that the commensurate ground state accounts well for the powder average magnetic scattering when included in its computation (**c**). Without the Y_3_^−^(σ) distortion, rods of diffuse scattering form **b** indicating short range correlations and the absence of long range order due to the frustration of the *J*_4_ and *J*_5_ exchange paths. **d** shows the good agreement of the diffuse scattering at 300 K with the simulation suggesting the presence of fully formed trimeron units well above *T*_N_.

Direct MC simulations were conducted assuming the six superexchange interactions described above. In the model the main exchange paths are the ferromagnetic *J*_3_ interaction and the antiferromagnetic *J*_1_, *J*_2_ and *J*_6_. If single values are assumed for all the *J*_4_ and *J*_5_ interactions, the MC simulation of the magnetic scattering clearly indicates the development of short range correlations leading to diffuse features (Fig. 6b, Supplementary Fig. 1) characterised by rods of scattered intensity centred at the half-integer position in *H*, integer *L* and elongated along *K*. The spin configuration obtained consists of fully formed ferromagnetic trimeron units antiferromagnetically correlated along *a*, that present short range correlations along the *b* and *c* axes. The experimentally observed ground state can only be reached by assuming an imbalance between the members of each pair of the two frustrated exchange paths *J*_4_ and *J*_5_. The Y_3_^−^(σ) distortion splits each pair of these interactions into two inequivalent ones (*J*_4_, *J*_4_′ and *J*_5_, *J*_5_′) alternating along the *a*-axis direction. Good agreement with the experimental data can be achieved in the simulations by fixing the *J*_3_ ferromagnetic interaction to unity and with the following set of antiferromagnetic *J*′s: *J*_1_ = *J*_2_ = *J*_6_ = 0.75, *J*_4_ = 0.5, *J*_4_′ = 0.3 and *J*_5_ = 0.1, *J*_5_′ = 0.05. The calculated magnetic scattering now shows sharp peaks (Fig. 6a and Supplementary Fig. 1) indicating the presence of 3D long range ordering. Computation of the powder average magnetic scattering using these relative values of the coupling constants shows excellent agreement with the experimental data (Fig. 6c). Inelastic neutron scattering investigations on single crystal samples would be required to probe the absolute values of the coupling constants.

**Fig. 7:**
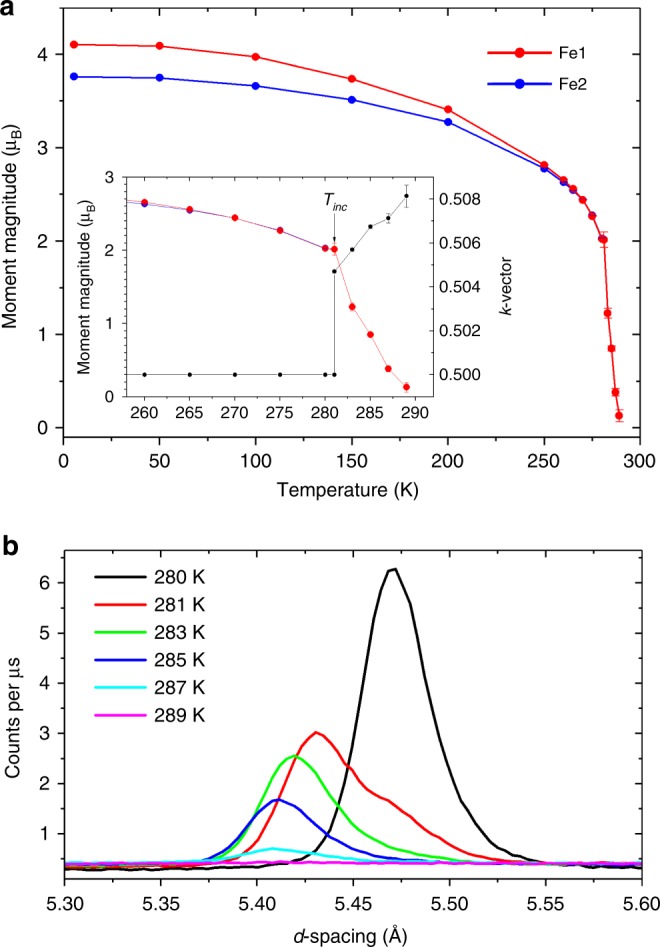
Temperature dependence of the magnetic ordering in CaFe_3_O_5_. **a** Ordered magnetic moment of the Fe1 and Fe2 sites in CaFe_3_O_5_ versus temperature from Rietveld refinement of the magnetic structure against neutron diffraction data. The inset of **a** shows the same data in the 260–290 K range alongside the magnetic ordering vector *k*, which becomes incommensurate at 281 K. **b** Evolution of the (½ 0 1) peak at 280 K collected on the 90° bank of the WISH diffractometer^23^ is shown on warming through the commensurate-to-incommensurate transition (see also Supplementary Fig. 4).

The moments refined for the Fe1 and Fe2 sites shown in Fig. 7a are similar in magnitude within the uncertainty of the refinement at 250 K, but gradually diverge to give saturated values at 5 K of 4.124(4) and 3.792(5) µ_B_, respectively. Bond valence sums derived from the fits to the neutron diffraction data also suggest a higher oxidation state for the Fe1 site than the Fe2 site with values of 2.673(2) and 2.136(2), respectively, at 300 K, which diverge slightly at low temperature to values of 2.756(2) and 2.095(2) at 5.5 K, mirroring the behaviour of the CO phase of Hong et al. and contrary to their CA phase^13^, for which the bond valence sums converge at low temperature instead. These values indicate that the system shows a partial charge ordering, however, the difference in the refined ordered moments is too small to describe the average of the system as entirely Fe^3+^ on the Fe1 site and Fe^2+^ on the Fe2 site even at 5.5 K, which is consistent with the distribution of the minority spin electron across the Fe1 and Fe2 sites in the localised trimeron units^2^. The increased separation of the moment sizes at low temperature suggests an increasing degree of localisation of the minority spin on the central Fe2 ion in the trimeron units, which moves towards the description of Gerardin et al. derived from their Mössbauer study: the minority spin first being distributed among trimers^12^, with the system then becoming more formally Fe^3+^ and Fe^2+^ at low temperature.

The arrangement of spins persists between 5.5 and 280 K, but on warming to 281 K a commensurate-incommensurate transition occurs. The data at 281 K shows coexistence of the commensurate magnetic peaks with a set of incommensurate magnetic peaks, then on warming further to 283 K only the incommensurate magnetic reflections are present (Fig. 7b, Supplementary Fig. 4b). A propagation vector of (0.5058(1), 0, 0) was deduced from the Rietveld refinements at 283 K Supplementary Fig. 4a, indicating the **k**-vector remains along the Σ(α00) symmetry line. The incommensurate structure was modelled with a spin density wave (SDW) propagating along the *a* axis, described in the *Cmcm1’(α00)00ss* magnetic superspace group, corresponding to the mΣ_3_ irreducible representation with order parameter direction (ξ, 0). At 283 K the separately refined amplitudes of the spin density wave are 1.98(4) and 2.08(3) µ_B_ for the Fe1 and Fe2 sites, respectively. Given that the difference between the Fe1 and Fe2 SDW amplitudes is within 2σ and the refined moments in the commensurate region converge on warming, both sites were constrained to have the same SDW amplitude in the incommensurate region, (refined value of 2.02(2) μ_B_). The phase coexistence between the commensurate and incommensurate phase that is observed at 281 K is expected since the lock-in character of the transition (see below) requires it to be first order. At 281 K the model has 55% of the incommensurate phase with propagation vector (0.5047(7) 0 0) and 45% of the commensurate phase with propagation vector (½ 0 0), assuming both phases have the same average moment. The incommensurate-regime is only adopted in a narrow temperature range, with a monotonic increase of the **k**-vector with increasing temperature to a maximum of *α* = 0.507(2) at 287 K before the moment falls to zero within error on warming to 289 K. The narrow temperature range for this incommensurate region presumably explains why it was not reported by Hong et al.^13^.

**Fig. 8:**
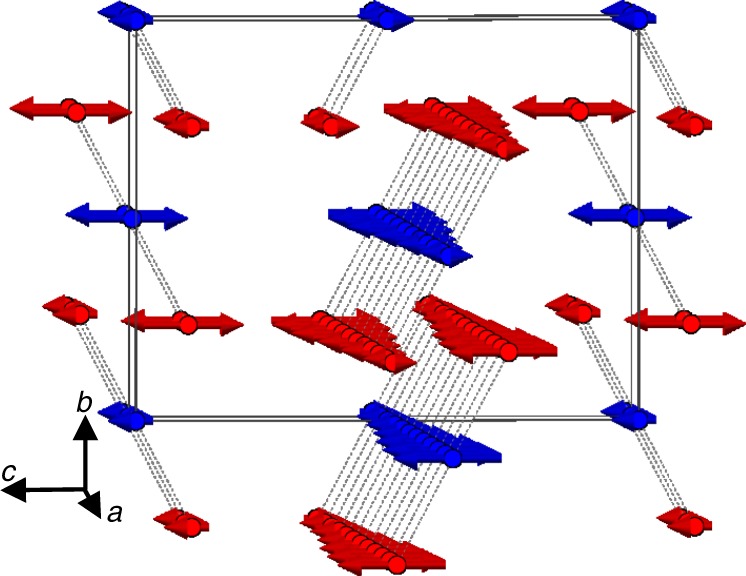
Incommensurate magnetic structure of CaFe_3_O_5_. Schematic of the modulation of the moment in the incommensurately ordered regime of CaFe_3_O_5_ (CO) with moments joined by dotted lines representing the trimerons. The *k*-vector has been exaggerated slightly to ~0.52 to shorten the period of the wave along *a* for ease of display. (Fe1: red, Fe2: blue).

A schematic representation of the incommensurate structure is shown in Fig. 8. *J*_1_, *J*_2_ and *J*_6_ are still net antiferromagnetic but the moment is described by a SDW along the *a* axis. The modulation is out of phase between the positions in the unit cell at 0 and ½ along *a*, which are the positions coupled by the frustrated *J*_4_ and *J*_5_ interactions. In contrast with the low temperature commensurate regime where a displacive distortion lifts the exchange degeneracy, in this incommensurate magnetic ordering regime, where coupling to a displacive distortion is not possible in principle, the magnetic frustration is resolved instead by modulating the moment along the *a*-axis at the expense of the entropic energy term. The result is that when one moment is maximised, its *J*_4/5_ coupled partner is minimised and vice versa, a situation comparable to that in the Fe^2+^ oxide selenide Sr_2_Fe_3_Se_2_O_3_ where the low temperature ordered structure involves inherently frustrated pathways which likewise become out of phase by adopting a modulated magnetic structure in a narrow temperature range close to the magnetic ordering temperature^21^. In this modulated model for CaFe_3_O_5_, the trimeron units are fully maintained in the incommensurate structure as shown by the dashed lines in Fig. 8, each iron centre having a moment that is fully ferromagnetically aligned and of equal magnitude to the other centres coupled to it by *J*_3_. The trimerons participate in the SDW as units, each one modulating its moment along the *a* axis.

The sharp Bragg peaks of the magnetic structure are lost at and above 289 K, with the majority of the magnetic scattering giving a uniformly increased background. However, the strongest peaks are still visible as diffuse scattering in the diffraction pattern at 300 K as shown for the (½ 0 1) reflection in Fig. 6d and Supplementary Fig. 2. The magnetic scattering can be well described by the frustrated model used for the calculation of Fig. 6b and Supplementary Figure 1 (right hand side). Figure 6d shows the agreement between the experimental data and the powder average calculated from the MC simulations with a single value for each of the pairs of *J*_4_ and *J*_5_ interactions. The good agreement clearly indicates that the trimeron units start to form and interact well above *T*_N_ and likely when the anomaly of the cell parameters appears around 400 K.

## Discussion

Hong et al. report phase separation in CaFe_3_O_5_ into phases that show CO and CA^13^, both with sharp nuclear and magnetic Bragg peaks indicating that they both present long range order. While the quality of their data shows that this is indeed the behaviour of their sample, we show here that this is not the intrinsic behaviour of stoichiometric CaFe_3_O_5_ which remains a single crystallographic phase, and shows only a single long range magnetically ordered phase (the CO phase with Fe^2+^/Fe^3+^ charge order) below the magnetic ordering transition.

Our refinements show that our sample is stoichiometric within the experimental uncertainty, while the refinements of Hong et al. show that their sample is slightly Fe rich^13^, with additional Fe ions occupying the Ca site at the level of about 4% giving a composition of Ca_0.952(8)_Fe_3.040(8)_O_5_. Neutron diffraction shows good contrast between Ca and Fe (bound scattering lengths of 4.9 and 9.5 fm, respectively), so the results suggest that there is indeed a small compositional difference between the samples. We presume that, as Hong et al. suggest^13^, this small degree of non-stoichiometry enables the CA phase to develop and compete effectively with the CO phase. The diffraction method probes the average structure and we suspect it is not possible to refine separately the compositions of the coexistant CA and CO phases, but we speculate that the CA portion of Hong et al.’s sample^13^ may be more Fe-rich than the CO phase.

Our several samples show different behaviour in the magnetic susceptibility with evidence that the phase fraction of the CA phase is proportional to the magnitude of a ferromagnetic signal in the magnetic susceptibility. This is also supported by the magnetic symmetry analysis of the two ordered phases. The CO phase orders adopting the *P*_*a*_*bca* magnetic space group that does not allow, by symmetry, a ferromagnetic moment in any direction. Instead the CA phase, as reported by Hong et al.^13^, orders with a **k** = (0, 0, 0) propagation vector and adopts the *Cm’cm’* magnetic space group (mΓ_4_^*+*^) which allows a ferromagnetic moment along the *b* direction, resulting in the observation of a spontaneous moment only in samples containing some of the CA phase. Clearly the synthesis of CaFe_3_O_5_ may result in a range of compositions. While our synthesis with the sample contained in an alumina crucible within a sealed silica tube enables us to make stoichiometric CaFe_3_O_5_, it is possible that higher temperature reactions may result in leaching of Ca from the sample into the silica tube.

**Fig. 9:**
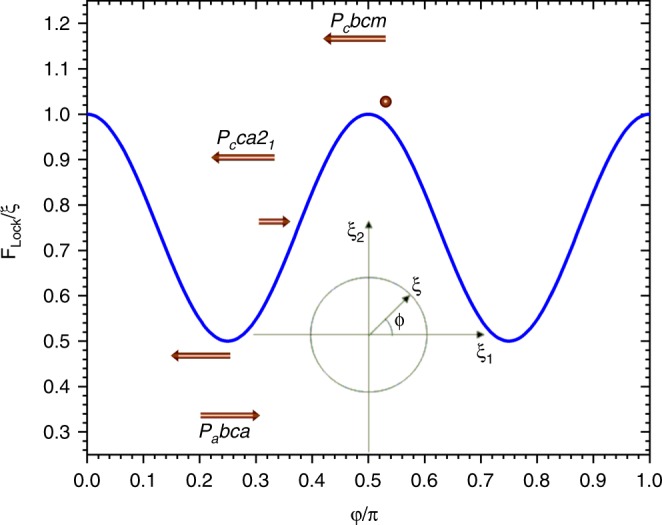
Phase dependence of the *F*_Lock_ = *ξ*_1_^4^ + *ξ*_2_^4^ invariant. The invariant is responsible for the lock in transition and the magnetic phase selection. The arrows indicate the stacking and the relative total moment of the trimeron units along the *b* directions for the different possible values of the magnetic phases (π/4, general choice and π/2 going from left to right) corresponding to the different space groups described in the text.

Our investigations on the stoichiometric sample showing only the CO phase show a narrow region of previously unseen incommensurate magnetic order from 289 K down to a commensurate ordering transition at 281 K. As observed experimentally, the propagation vector jumps from (0.5047(7), 0, 0) to the commensurate value (½, 0, 0), remaining along the Σ symmetry line (α00). It is worth underlining that **k** *=* (½, 0, 0) is not a special point of the Brillouin zone due to the *C* centring of the lattice, and the low temperature magnetic structure can be described as a commensurate spin density wave with the magnetic phase locked to the *ϕ* = π/4 value. The lock-in of the propagation vector to the commensurate value and the selection of the magnetic phase can be understood considering the symmetry of the transition. As we show in the Results section both the incommensurate and commensurate magnetic phases are described by the two dimensional order parameter (ξ_1_, ξ_2_) transforming as the mΣ_3_ irreducible representation of the parent *Cmcm* space group. Considering this order parameter, the Landau free energy, describing the magnetic transition, contains a fourth degree lock-in invariant *F*_Lock_ = *ξ*_1_^4^ + *ξ*_2_^4^. This term is allowed only when the propagation vector is locked to the commensurate (½, 0, 0) value and the energy gain deriving from its activation explains the lock-in transition. The magnetic phase selection of the spin density wave can be understood from the phase dependence of the *F*_Lock_ term in the (*ξ*_1_, *ξ*_2_) plane. This dependence is shown in Fig. 9 and shows a minimum for *ϕ* = π/4. This point corresponds to the order parameter direction (*ξ*, *ξ*), and describes the magnetic structure observed experimentally in the *P*_*a*_*bca* magnetic space group. The maxima at *ϕ* = 0 and *ϕ* = π/2 represent the solution with only half of the Fe sites ordered (half the trimerons are ordered and the other half have zero moment (represented by the dot in Fig. 9)), corresponding to the *P*_*c*_*bcm* magnetic space group with order parameter direction (*ξ*, 0), whereas a general point (*ξ*_1_, *ξ*_2_) with *ξ*_1_ ≠ *ξ*_2_ ≠ 0 represents the *P*_*c*_*ca*2_1_ solution (in which the trimerons carry different moments). Since the moduli of the neutron structure factors are insensitive to the phase choice, this explains why the last two solutions gave comparable fits in the Rietveld refinements (see above). Moreover the lock-in of the propagation vector to the commensurate value also allows the coupling of the magnetic order parameter (*ξ*_1_, *ξ*_2_) to that of the Y_3_^−^ displacive distortion (σ) through the coupling term σ*ξ*_1_*ξ*_2_, which breaks the exchange frustration allowing for the long range ordered structure through the spin Jahn-Teller effect.

Our observations of diffuse magnetic scattering above *T*_N_ and non-linear behaviour of the lattice parameters as a function of temperature well above *T*_N_ are evidence of short range exchange interactions being influential in CO-CaFe_3_O_5_ well above the Néel temperature. The MC simulations indicate the formation of the ferromagnetic trimeron units in this temperature range above *T*_N_. The structure of the diffuse scattering and the MC simulations suggest that these units present short range correlations at RT, and the study of their dynamics merit further investigation. Gerardin et al. originally proposed that the mechanism of electron exchange to average the Fe^3+^ and Fe^2+^ charges above *T*_N_ was confined to an Fe^2+^O_6_ octahedron and two neighbouring Fe^3+^O_6_ octahedra with which it shares edges^12^. They referred to this as a trimer, and describe the spin exchange as localised in these trimers up to at least 400 K before eventually becoming fully delocalised. In contrast, in Fe_3_O_4_^2^, the trimerons form when the system already shows magnetic long range order, accompanied by much larger changes in Fe–Fe distances than are observed in the CO phase of CaFe_3_O_5_ reported here and in ref. ^13^.

In conclusion, CaFe_3_O_5_ shows CO below the magnetic ordering transition with localisation of the minority spin electron in so-called trimeron units first proposed for Fe_3_O_4_^2^. Long range electronic phase separation is not found in the stoichiometric compound with a 2:1 ratio of Fe^3+^ and Fe^2+^ ions, but Hong et al. importantly show that the development of an alternative antiferromagnetic phase, the CA phase without charge ordering and without trimeron formation, occurs when the sample is slightly non-stoichiometric^13^. Our results, taken together with those of Hong et al.^13^ and the literature on related compounds underline the subtleties of these mixed-valent phases and suggest that targeted further tuning of the competition between electronic states through adjustment of the Ca:Fe ratio, control of the oxide content and control of the microstructure will be fruitful approaches for subtly controlling the physical properties of these oxides.

## Methods

### Synthesis

Polycrystalline samples of CaFe_3_O_5_ were synthesised from stoichiometric amounts of CaO, (Alfa Aesar 99.95%) Fe_2_O_3_ (Alfa Aesar 99.998%), and Fe (Alfa Aesar 99.998%). The reactants were ground together inside an argon-filled dry glovebox using an agate pestle and mortar. The ground powder was pressed into a pellet, placed inside an alumina crucible, and sealed inside an evacuated silica ampoule. The product was found to form in high phase purity with repeated annealings between 800 and 1050 °C. Higher synthesis temperatures led to higher quantities of impurities and discolouration of the alumina crucible. Multiple samples were synthesised, as detailed in Supplementary Table 1 which all appear to be majority charge-ordered CaFe_3_O_5_, with subtle differences as described in the body of the paper.

### Diffraction measurements

High resolution X-ray Powder Diffraction measurements for structure solution and analysis were performed on beamline I11^22^ at the Diamond Light Source Ltd, UK using the high resolution MAC detector at room temperature, 100 and 500 K with temperature controlled by a nitrogen cryostream. The temperature dependence of the lattice parameters was measured using the high count rate PSD detector to continuously collect patterns on cooling the sample from 500 to 100 K, with a pattern collected every 1.5 K on average. Neutron Powder Diffraction measurements were performed from 5 to 300 K on the WISH instrument^23^ at the ISIS Pulsed Neutron and Muon Facility, UK with the samples contained in indium-sealed thin-walled vanadium cylinders inside a closed-cycle refrigerator (CCR). Structure solution and Rietveld refinements were performed using the TOPAS Academic software version 6^17,18^, for the nuclear and commensurate magnetic structure and FullProf^24^ was used to refine the incommensurate magnetic structure. Tables of crystallographic data are given in Supplementary Tables 2–7.

### Magnetometry

Measurements were carried out using a Quantum Design MPMS-XL SQUID magnetometer. The DC susceptibility was measured by cooling the sample to 2 K in zero applied field (ZFC) measuring the magnetisation on warming to 330 K in a 1000 Oe field, then cooling back down in the applied field (FC) and measuring again on warming, Magnetisation isotherms (–5 ≤ *μ*_0_*H*/T ≤ 5) were collected by first applying a field of 5 T at 300 K, then cooling to the measurement temperature in the field, then measuring the magnetisation on sweeping the field in steps down to −5 T and back to +5 T. AC magnetometry measurements were carried out at frequencies of 1, 10, 100 and 1000 Hz in a DC field of 3 Oe and an oscillating field of amplitude 3.5 Oe. The 1000 Hz data contained a high level of noise and are only shown in Supplementary Figure 3. Samples were contained in gelatin capsules.

### Monte Carlo simulations

Classical Monte Carlo simulation was performed using an in-house code^25^ based on a Metropolis algorithm^26^. A 20 × 20 × 20 supercell of the structural unit cell was used and the energy minimisation used Ising spins on each Fe site and the exchange interactions described in the text with cyclic boundary conditions.

## Supplementary information

Supplementary Information

## Data Availability

The datasets generated during and/or analysed during the current study are available from the corresponding author on reasonable request. Correspondence should be addressed to S.J.Clarke (simon.clarke@chem.ox.ac.uk).
