# Supplementary material for: Single phase charge ordered stoichiometric CaFe3O5 with commensurate and incommensurate trimeron ordering
Source: Nat Commun. 2019 Dec 2;10:5475. doi: 10.1038/s41467-019-13450-5 (PMC6889228; doi:10.1038/s41467-019-13450-5)
Supplement: Supplementary file 1 — Supplementary Information [file 41467_2019_13450_MOESM1_ESM.pdf]

Single Phase Charge Ordered Stoichiometric $CaFe_3O_5$ with Commensurate and Incommensurate Trimeron Ordering

Cassidy et al.

Supplementary Information

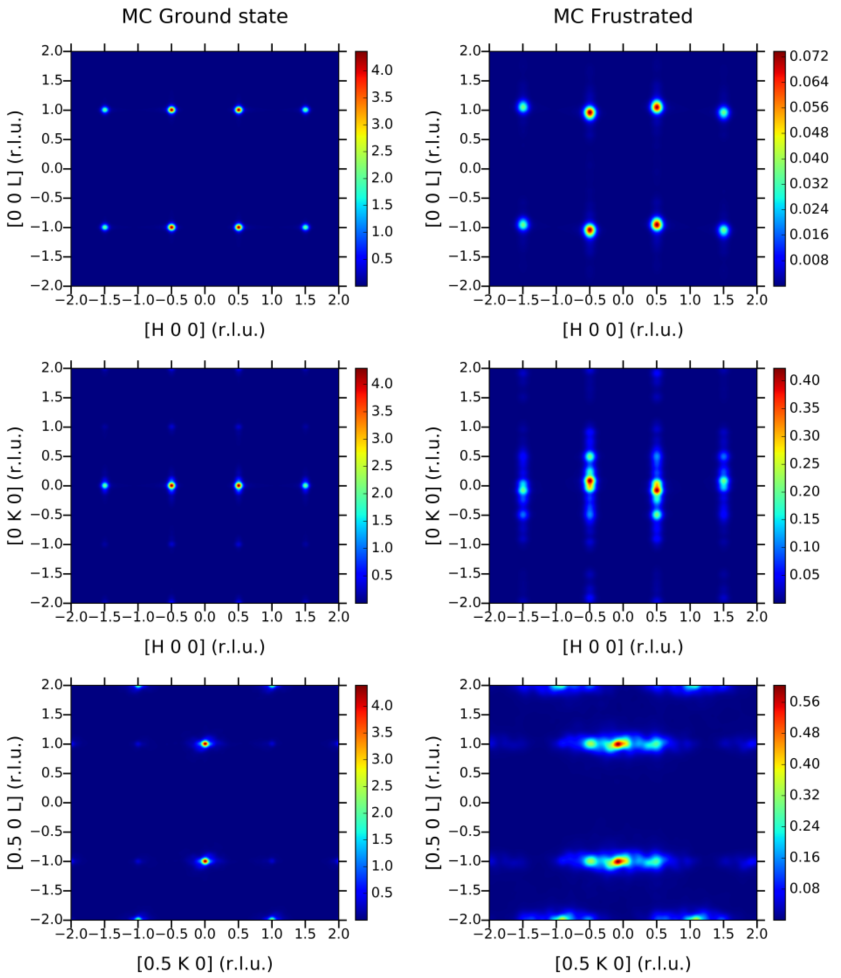

Supplementary Figure 1. Magnetic scattering cross sections calculated from the MC simulation for $CaFe_3O_5$. Left side: H0L, HK0 and 0.5KL scattering planes calculated from the MC simulations taking into account the $Y_3^-(\sigma)$ distortion that relieves the frustration of the J_4 and J_5 exchange paths. Right side: H0L, HK0 and 0.5KL scattering planes calculated from the MC simulations without the $Y_3^-(\sigma)$ distortion showing the clear presence of rods of diffuse scattering indicating short range correlations and the absence of long range ordering. The Bottom panel is repeated in Figure 6 of the main document.

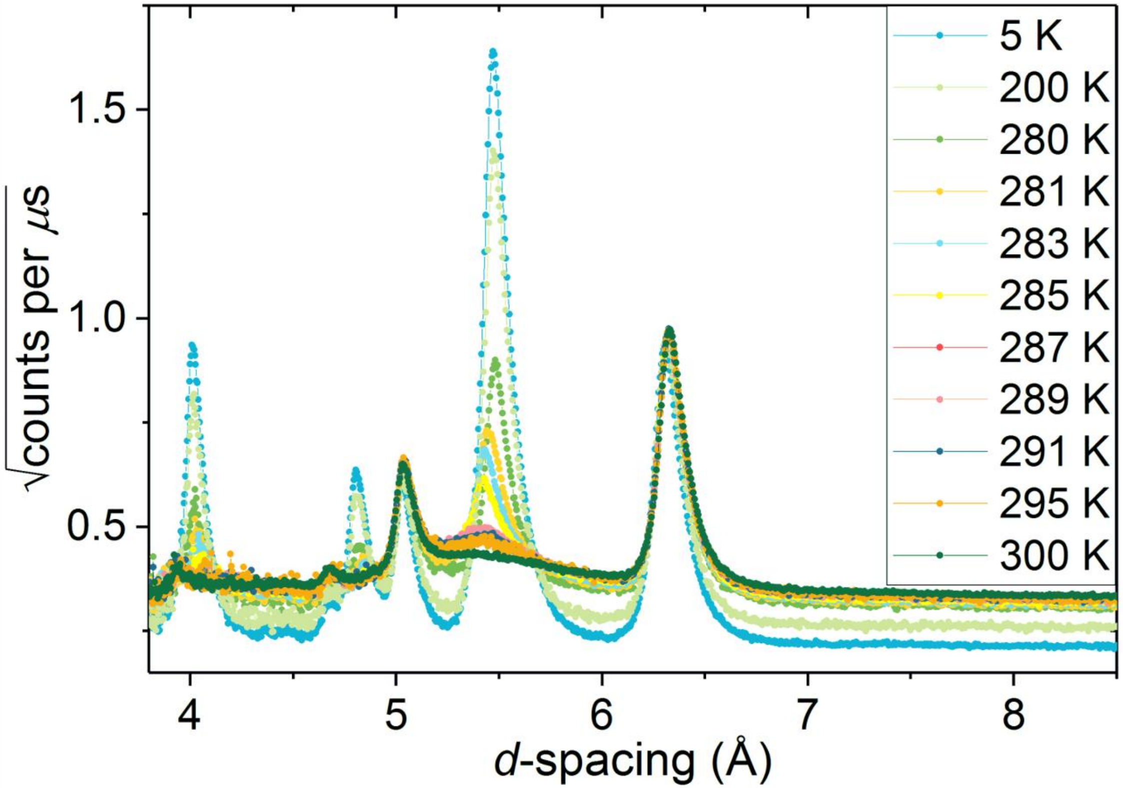

Supplementary Figure 2. Neutron powder diffraction of charge ordered $CaFe_3O_5$ (sample **A**) from the 27° Bank of the WISH diffractometer. The magnetic (½ 0 1) peak at ~5.5 Å is no longer a sharp Bragg peak above 289 K but can be seen to still give a diffuse feature in this region that persists above 300 K. The intensity is shown on a square root scale to highlight this scattering.

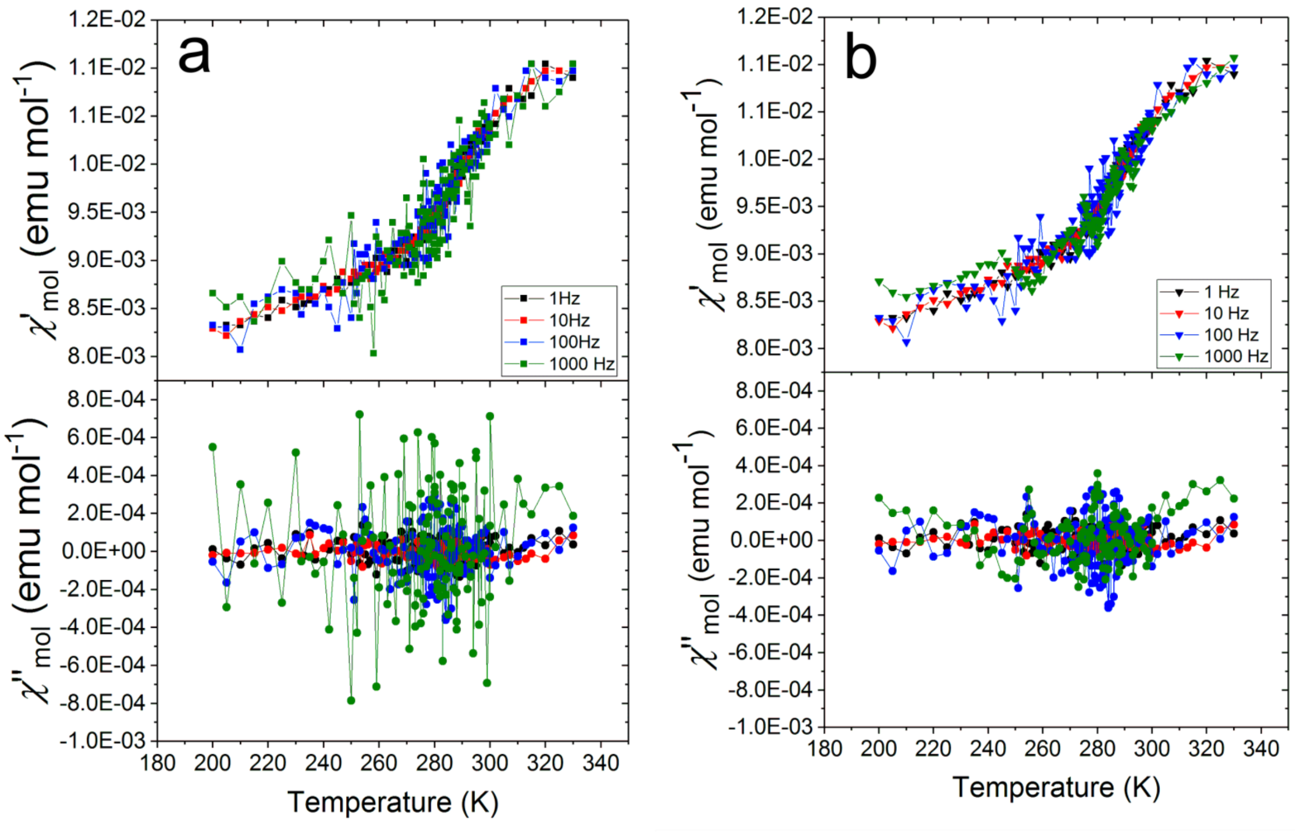

Supplementary Figure 3. Real and imaginary parts of the AC susceptibility of sample **A**, as shown in Figure 4 with the addition of the 1000 Hz data. The 1000 Hz data contained a large amount of noise relative to the signal. **a** shows all the data as measured **b** shows the 1000Hz data smoothed by adjacent point averaging with a sample size of 5.

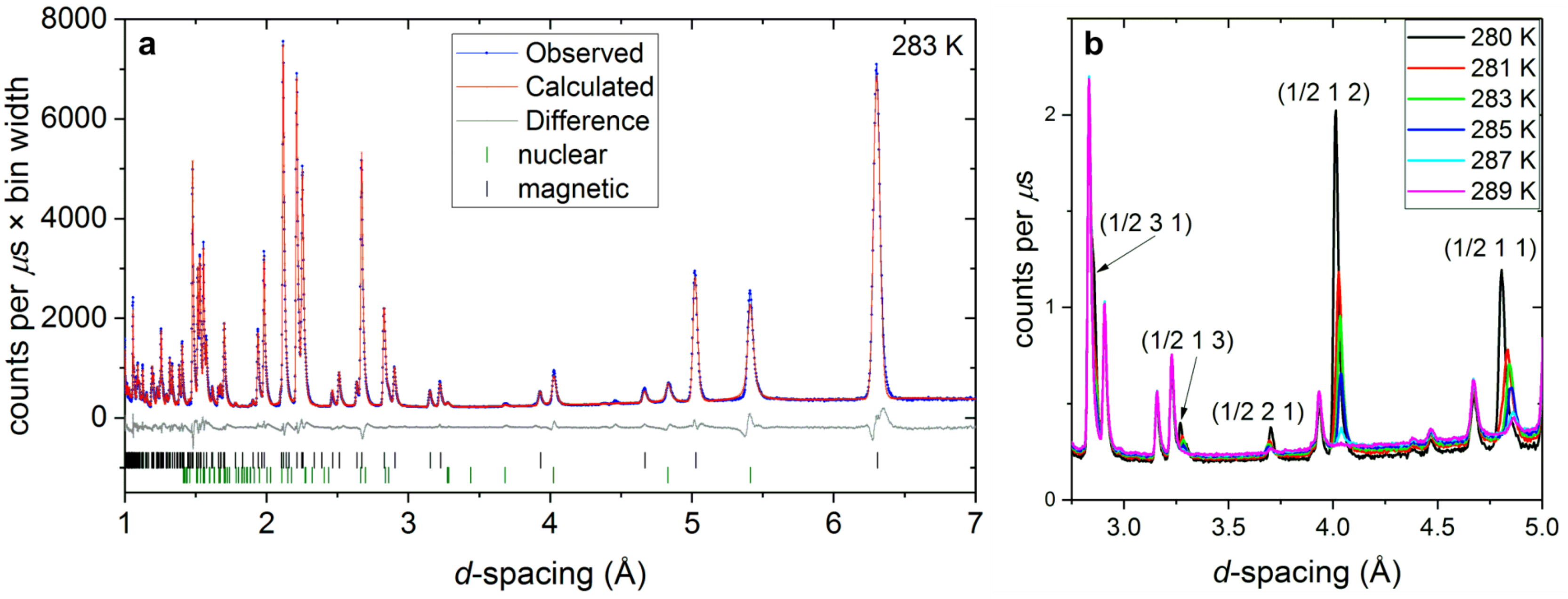

Supplementary Figure 4 a) Rietveld refinement of sample A in the incommensurate magnetic order regime at 283 K shown against the 90° detector bank of the WISH neutron diffractometer. Refinement performed using FullProf. **b)** magnetic reflections over the incommensurate region, in addition to the ½ 0 1 shown in Figure 7b in the main document.

Supplementary Table 1 Description of sample syntheses.

Sample	Procedure	% CA by XRD at 100 K	χ_{mol}(260 K)- χ_{mol}(300 K) / emu mol^{-1}
A	1st step: 10°C min^{-1} to 1000°C, annealed for 43 h, then the furnace was switched off and the sample allowed to cool inside the furnace. 2nd step: 10°C min^{-1} to 800°C, annealed for 88 h, then the furnace switched off and the sample allowed to cool inside the furnace.	<1 %	<0
B	1st step: 10°C min^{-1} to 1050°C, annealed for 72 h, cooled at 0.5°C min^{-1} to room temperature.	2.71(5) %	0.028
C	1st step: 10°C min^{-1} to 900°C, annealed for 82 h, then the furnace was switched off and the sample allowed to cool inside the furnace.	<1 %	<0
D	1st step: 10°C min^{-1} to 1000°C, annealed for 24 h, quenched. 2nd step: 10°C min^{-1} to 1000°C, annealed for 62 h, quenched.	1.80(6) %	0.007
E	1st step: 10°C min^{-1} to 900°C, annealed for 24 h, quenched. 2nd step: 10°C min^{-1} to 900°C, annealed for 65 h, quenched.	<1 %	0.002

Supplementary Table 2. Ambient temperature crystal structure

Instrument	WISH
Temperature (K)	300
Rwp (global)	3.41
a / Å	3.03497(2)
b / Å	10.04862(9)
c / Å	12.63418(11)
V / Å^3	385.308(6)
Space group	*Cmcm*

Atom	Site	*x*	*y*	*z*	Occupancy	U_{11}	U_{22}	U_{33}	U_{23}
Ca(/ Fe)	4*c*	0	0.5192(1)	0.25	1.000/0.000(3)	0.0122(11)	0.0015(11)	0.0135(10	0
Fe1(/Ca)	8*f*	0	0.26578(6)	0.11106(4)	1.000/0.000(3)	0.0050(4)	0.0029(4)	0.0052(3)	0.0001(3)
Fe2(/Ca)	4*a*	0	0	0	0.997/0.003(4)	0.0085(6)	0.0092(6)	0.0065(5)	0.0005(4)
O1	4*c*	0	0.1924(1)	0.25	1	0.0014(7)	0.0125(10)	0.0023(8)	0
O2	8*f*	0	0.64702(9)	0.44795(7)	1	0.0140(6)	0.0111(6)	0.0116(5)	0.0042(5)
O3	8*f*	0	0.9024(1)	0.13542(6)	1	0.0115(6)	0.0111(6)	0.0049(6)	0.0044(5)

Supplementary Table 3. Low temperature magnetic structure

Instrument	WISH					
Temperature (K)	5					
a / Å	6.05990(7)					
b / Å	10.04302(11)					
c / Å	12.58747(15)					
Space group	P_abca (61.438)					
Site	*x*	*y*	*z*	M_x /μ_B	M_y /μ_B	M_z /μ_B
Fe1	0.87500	0.01538(5)	0.11094(4)	0	0	4.124(4)
Fe2	0.12500	0.25000	0.00000	0	0	3.792(5)

Supplementary Table 4. Incommensurate magnetic structure

Instrument	WISH
Temperature (K)	283
a / Å	3.03413(4)
b / Å	10.05606(14)
c / Å	12.61796(16)
Irrep	mSM3
order parameter	(ξ,0)
**k**-active	(0.50578(8), 0, 0)
Super space group	*Cmcm1'(α00)00ss*

Site	*x*	*y*	*z*	A_x(max) /μ_B	A_y(max) /μ_B	A_z(max) /μ_B
Fe1	0	0.26568(7)	0.11075(4)	0	0	2.02(2)
Fe2	0	0	0	0	0	2.02(2)

Lattice parameters were fixed at values for 280 K nuclear structure. The SDW amplitudes of the Fe1 and Fe2 sites were confined to be the same. Amplitudes reported here are the maxima, related to the average moment size by ~0.637**A*(max)

Temperature (K)	283	285	287	289
**k**-vector	0.50578(8)	0.50674(8)	0.5070(2)	0.5073(8)
SDW amplitude (max) along *c* /μ_B	2.02(2)	1.39(2)	0.71(3)	0.25(5)

Supplementary Table 5. Refined structural parameters as a function of temperature from neutron powder diffraction.

Parameter	WISH 5.5 K sample A	WISH 50 K sample A	WISH 100 K sample A	WISH 150 K sample A	WISH 200 K sample A	WISH 250 K sample A	WISH 260 K sample A	WISH 265 K sample A
R_{wp} / %	3.47	3.64	3.56	3.59	3.69	3.59	3.59	3.66
a / Å	3.02991(4)	3.03000(4)	3.03036(4)	3.03110(4)	3.03205(4)	3.03337(4)	3.03352(4)	3.03367(4)
b / Å	10.04287(12)	10.04290(12)	10.04345(12)	10.04616(12)	10.05010(12)	10.05546(12)	10.05576(12)	10.05616(12)
c / Å	12.58731(15)	12.58768(16)	12.58974(15)	12.59437(16)	12.60018(16)	12.60942(15)	12.61111(15)	12.61242(15)
V / Å^3	383.019(8)	383.043(8)	383.172(8)	383.510(8)	383.957(8)	384.611(8)	384.694(8)	384.769(8)
Ca *y*	0.51854(12)	0.51859(13)	0.51864(12)	0.51871(12)	0.51899(12)	0.51937(11)	0.51949(11)	0.51926(11)
Fe1 *y*	0.26593(5)	0.26587(5)	0.26587(5)	0.26582(5)	0.26579(5)	0.26572(5)	0.26573(5)	0.26571(5)
O1 *y*	0.19211(10)	0.19209(10)	0.19206(10)	0.19213(10)	0.19237(10)	0.19227(9)	0.19225(8)	0.19223(8)
O2 *y*	0.64881(8)	0.64875(8)	0.64880(8)	0.64896(8)	0.64880(8)	0.64876(7)	0.64861(7)	0.64860(7)
O3 *y*	0.90108(9)	0.90103(9)	0.90127(9)	0.90144(8)	0.90154(8)	0.90189(7)	0.90194(7)	0.90197(7)
Fe1 *z*	0.11082(4)	0.11080(4)	0.11082(4)	0.11078(4)	0.11067(4)	0.11068(3)	0.11069(3)	0.11074(3)
O2 *z*	0.44729(7)	0.44735(8)	0.44724(7)	0.44717(7)	0.44719(7)	0.44712(6)	0.44716(6)	0.44710(6)
O3 *z*	0.13556(6)	0.13551(6)	0.13556(6)	0.13552(6)	0.13556(6)	0.13567(5)	0.13567(5)	0.13568(5)
U_{iso} Ca / Å^2	0.0037(4)	0.0041(4)	0.0043(4)	0.0050(4)	0.0065(4)	0.0075(4)	0.0075(4)	0.0073(4)
U_{iso} Fe1 / Å^2	0.00141(15)	0.00135(16)	0.00181(15)	0.00251(15)	0.00283(15)	0.00347(14)	0.00374(14)	0.00378(14)
U_{iso} Fe2 / Å^2	0.0046(2)	0.0048(3)	0.0053(2)	0.0061(2)	0.0064(2)	0.0079(2)	0.0082(2)	0.0085(2)
U_{iso} O1 / Å^2	0.0016(4)	0.0018(4)	0.0019(4)	0.0025(3)	0.0030(3)	0.0039(3)	0.0034(3)	0.0033(3)
U_{iso} O2 / Å^2	0.0074(3)	0.0073(3)	0.0078(3)	0.0080(3)	0.0089(3)	0.0096(2)	0.0100(2)	0.0096(2)
U_{iso} O3 / Å^2	0.0071(2)	0.0070(2)	0.0073(2)	0.0078(2)	0.0081(2)	0.00831(19)	0.00845(18)	0.00842(19)
Fe1 μ_B (*z*)	3.768(5)	3.747(5)	3.671(5)	3.519(5)	3.278(5)	2.780(5)	2.631(5)	2.551(5)
Fe2 μ_B (*z*)	4.114(5)	4.092(5)	3.985(5)	3.742(4)	3.412(4)	2.817(4)	2.655(4)	2.562(4)

Cmcm structural model as in Supplementary Table 2

Supplementary Table 5 (continued). Refined structural parameters as a function of temperature from neutron powder diffraction.

Parameter	WISH 270 K sample A	WISH 275 K sample A	WISH 280 K sample A	WISH 300 K Sample A
R_{wp} / %	3.75	3.74	3.73	3.55
a / Å	3.03377(4)	3.03399(4)	3.03413(4)	3.03497(4)
b / Å	10.05613(12)	10.05635(13)	10.05606(13)	10.04860(13)
c / Å	12.61356(16)	12.61567(16)	12.61796(16)	12.63425(16)
V / $Å^3$	384.815(8)	384.915(8)	384.991(8)	385.309(8)
Ca *y*	0.51957(11)	0.51946(11)	0.51954(11)	0.51980(10)
Fe1 *y*	0.26564(5)	0.26566(5)	0.26574(5)	0.26584(5)
O1 *y*	0.19230(9)	0.19235(9)	0.19224(8)	0.19213(8)
O2 *y*	0.64854(7)	0.64851(7)	0.64832(7)	0.64702(6)
O3 *y*	0.90206(7)	0.90206(7)	0.90218(7)	0.90273(7)
Fe1 *z*	0.11072(3)	0.11072(3)	0.11072(3)	0.11093(3)
O2 *z*	0.44723(6)	0.44730(6)	0.44728(6)	0.44779(6)
O3 *z*	0.13559(5)	0.13567(5)	0.13569(5)	0.13553(5)
U_{iso} Ca / $Å^2$	0.0075(4)	0.0079(4)	0.0084(4)	0.0101(3)
U_{iso} Fe1 / $Å^2$	0.00367(14)	0.00405(14)	0.00383(13)	0.00404(13)
U_{iso} Fe2 / $Å^2$	0.0081(2)	0.0087(2)	0.0085(2)	0.0087(2)
U_{iso} O1 / $Å^2$	0.0036(3)	0.0043(3)	0.0035(3)	0.0045(3)
U_{iso} O2 / $Å^2$	0.0097(2)	0.0100(2)	0.0102(2)	0.0115(2)
U_{iso} O3 / $Å^2$	0.00839(19)	0.00879(19)	0.00873(18)	0.00938(17)
Fe1 μ_B (*z*)	2.447(5)	2.280(5)	2.029(6)	
Fe2 μ_B (*z*)	2.447(4)	2.277(4)	2.025(5)	

Cmcm structural model as in Supplementary Table 2

Supplementary Table 6. Bond lengths at different temperatures from neutron powder diffraction.

Parameter	WISH 5.5 K sample A	WISH 50 K sample A	WISH 100 K sample A	WISH 150 K sample A	WISH 200 K sample A	WISH 250 K sample A
Ca-O x 2 / Å	2.3095(12)	2.3090(12)	2.3086(12)	2.3091(12)	2.3097(11)	2.3072(10)
Ca-O x 4 / Å	2.4003(9)	2.4013(9)	2.4002(9)	2.4008(8)	2.4023(8)	2.4029(7)
Fe1-O x 1 / Å	1.9023(6)	1.9024(6)	1.9026(6)	1.9033(6)	1.9043(6)	1.9057(5)
Fe1-O x 2 / Å	2.0527(7)	2.0529(7)	2.0524(7)	2.0513(6)	2.0524(6)	2.0530(6)
Fe1-O x 2 / Å	2.0577(7)	2.0578(7)	2.0596(7)	2.0616(7)	2.0635(7)	2.0675(6)
Fe1-O x 1 / Å	2.2294(10)	2.2290(11)	2.2306(10)	2.2313(10)	2.2316(10)	2.2345(9)
Fe2-O x 2 / Å	1.9745(8)	1.9742(8)	1.9738(8)	1.9732(8)	1.9740(7)	1.9748(7)
Fe2-O x 4 / Å	2.2291(6)	2.2285(6)	2.2294(6)	2.2314(6)	2.2310(6)	2.2322(5)
Fe1-Fe1 (J_1) / Å & Fe2-Fe2 (J_2) / Å	3.02991(4)	3.03000(4)	3.03036(4)	3.03110(4)	3.03205(4)	3.03337(4)
Fe1-Fe2 (J_3) / Å	3.0130(5)	3.0124(5)	3.0128(5)	3.0130(5)	3.0133(5)	3.0145(5)
Fe1-Fe1 (J_4) / Å	3.1907(8)	3.1903(8)	3.1912(8)	3.1913(8)	3.1902(8)	3.1924(7)
Fe1-Fe2 (J_5) / Å	3.1252(4)	3.1256(4)	3.1260(4)	3.1270(4)	3.1278(4)	3.1301(4)
Fe1-Fe1 (J_6) / Å	3.5038(9)	3.5044(10)	3.5045(9)	3.5068(9)	3.5112(9)	3.5135(8)

Supplementary Table 6 (continued). Bond lengths at different temperatures from neutron powder diffraction.

Parameter	WISH 260 K sample A	WISH 265 K sample A	WISH 270 K sample A	WISH 275 K sample A	WISH 280 K sample A	WISH 300 K sample A
Ca-O x 2 / Å	2.3062(10)	2.3079(10)	2.3061(10)	2.3074(10)	2.3060(10)	2.3025(10)
Ca-O x 4 / Å	2.4035(7)	2.4023(7)	2.4041(8)	2.4032(7)	2.4031(7)	2.4038(7)
Fe1-O x 1 / Å	1.9059(5)	1.9055(5)	1.9053(5)	1.9055(5)	1.9065(5)	1.9068(5)
Fe1-O x 2 / Å	2.0542(6)	2.0542(6)	2.0547(6)	2.0555(6)	2.0570(6)	2.0685(6)
Fe1-O x 2 / Å	2.0678(6)	2.0682(6)	2.0691(6)	2.0693(6)	2.0696(6)	2.0716(6)
Fe1-O x 1 / Å	2.2350(9)	2.2366(9)	2.2355(9)	2.2350(9)	2.2360(9)	2.2394(8)
Fe2-O x 2 / Å	1.9748(7)	1.9749(7)	1.9736(7)	1.9747(7)	1.9746(6)	1.9716(6)
Fe2-O x 4 / Å	2.2311(5)	2.2314(5)	2.2305(5)	2.2302(5)	2.2290(5)	2.2182(5)
Fe1-Fe1 (J_1) / Å & Fe2-Fe2 (J_2) / Å	3.03352(4)	3.03367(4)	3.03377(4)	3.03399(4)	3.03413(4)	3.03497(4)
Fe1-Fe2 (J_3) / Å	3.0148(5)	3.0150(5)	3.0144(5)	3.0147(5)	3.0155(5)	3.0167(5)
Fe1-Fe1 (J_4) / Å	3.1930(7)	3.1943(7)	3.1940(8)	3.1945(7)	3.1951(7)	3.2033(7)
Fe1-Fe2 (J_5) / Å	3.1303(4)	3.1309(4)	3.1314(4)	3.1314(4)	3.1309(4)	3.1311(4)
Fe1-Fe1 (J_6) / Å	3.5137(8)	3.5128(8)	3.5136(8)	3.5142(8)	3.5149(8)	3.5141(8)

Supplementary Table 7. Refined structural parameters as a function of temperature for several samples from X-ray powder diffraction.

Parameter	I11 100 K sample A	I11 500 K sample A	I11 100 K sample B	I11 500 K sample B	I11 100 K sample C	I11 500 K sample C
R_{wp} / %	7.10	7.33	9.24	7.81	9.62	7.20
χ^2	2.10	2.01	2.39	2.02	2.56	1.96
a / Å	3.025920(5)	3.040020(5)	3.024920(7)	3.039522(6)	3.026137(8)	3.040265(7)
b / Å	10.032752(16)	10.018698(18)	10.02973(2)	10.015816(19)	10.03258(3)	10.01916(2)
c / Å	12.571867(19)	12.67327(2)	12.57117(3)	12.67398(3)	12.57204(3)	12.67377(3)
Volume / $Å^3$	381.6605(10)	385.9904(12)	381.3985(15)	385.8376(13)	381.6870(18)	386.0544(15)
Ca *y*	0.51981(7)	0.52012(8)	0.51973(8)	0.52014(8)	0.51998(9)	0.52022(8)
Fe1 *y*	0.26562(4)	0.26624(4)	0.26550(5)	0.26620(4)	0.26564(5)	0.26635(4)
O1 *y*	0.1941(2)	0.1932(2)	0.1945(3)	0.1946(2)	0.1943(3)	0.1935(2)
O2 *y*	0.65013(15)	0.64431(17)	0.64911(19)	0.64383(17)	0.6494(2)	0.64376(16)
O3 *y*	0.90176(16)	0.90303(17)	0.9015(2)	0.90346(16)	0.9013(2)	0.90309(16)
Fe1 *z*	0.10998(3)	0.11090(3)	0.11012(3)	0.11131(3)	0.10991(3)	0.11099(3)
O2 *z*	0.44853(12)	0.45012(14)	0.44805(16)	0.44963(14)	0.44828(15)	0.44909(13)
O3 *z*	0.13502(11)	0.13382(13)	0.13495(14)	0.13458(13)	0.13559(14)	0.13426(11)
U_{11} Ca / $Å^2$	0.0095(4)	0.0141(5)	0.0079(4)	0.0142(5)	0.0100(5)	0.0132(5)
U_{11} Fe1 / $Å^2$	0.00945(19)	0.0173(2)	0.0087(2)	0.0149(2)	0.0099(3)	0.0155(2)
U_{11} Fe2 / $Å^2$	0.0106(3)	0.0234(4)	0.0110(4)	0.0231(4)	0.0116(4)	0.0233(4)
U_{iso} O1 / $Å^2$	0.0093(4)	0.0144(6)	0.0077(6)	0.0151(6)	0.0102(6)	0.0163(6)
U_{iso} O2 / $Å^2$	0.0100(3)	0.0191(4)	0.0103(4)	0.0208(4)	0.0097(4)	0.0185(4)
U_{iso} O3 / $Å^2$	0.0092(3)	0.0164(4)	0.0080(4)	0.0159(4)	0.0082(4)	0.0141(4)
U_{22} Ca / $Å^2$	0.0097(4)	0.0198(5)	0.0081(5)	0.0170(5)	0.0076(5)	0.0188(5)
U_{22} Fe1 / $Å^2$	0.0083(2)	0.0158(3)	0.0068(3)	0.0136(3)	0.0064(3)	0.0141(3)
U_{22} Fe2 / $Å^2$	0.0094(3)	0.0185(4)	0.0061(3)	0.0148(3)	0.0071(4)	0.0172(4)
U_{33} Ca / $Å^2$	0.0093(3)	0.0220(5)	0.0072(4)	0.0281(6)	0.0065(4)	0.0208(4)
U_{33} Fe1 / $Å^2$	0.0095(2)	0.0162(2)	0.0064(2)	0.0194(3)	0.0059(2)	0.0151(2)
U_{33} Fe2 / $Å^2$	0.0092(3)	0.0163(3)	0.0074(3)	0.0185(4)	0.0064(3)	0.0147(3)
U_{23} Fe1 / $Å^2$	-0.00009(17)	0.0007(2)	0.0007(2)	0.0012(2)	0.0001(2)	0.0012(2)
U_{23} Fe2 / $Å^2$	0.0004(2)	0.0037(3)	0.0013(3)	0.0032(3)	0.0006(3)	0.0036(3)

Supplementary Table 7 (continued). Refined structural parameters as a function of temperature for several samples from X-ray powder diffraction.

Parameter	I11 100 K sample D	I11 500 K sample D	I11 100 K sample E	I11 500 K sample E
R_{wp} / %	8.26	7.13	7.85	7.21
χ^2	2.36	1.84	2.00	1.88
a / Å	3.025158(8)	3.039675(8)	3.025990(7)	3.040454(6)
b / Å	10.02958(3)	10.01614(3)	10.03071(2)	10.01749(2)
c / Å	12.57138(4)	12.67372(3)	12.57066(2)	12.67281(2)
Volume / Å^3	381.4288(19)	385.8616(18)	381.5551(14)	385.9850(13)
Ca *y*	0.51986(9)	0.52025(9)	0.51985(7)	0.52015(7)
Fe1 *y*	0.26550(5)	0.26622(4)	0.26545(4)	0.26628(4)
O1 *y*	0.1945(3)	0.1940(3)	0.1936(2)	0.1936(2)
O2 *y*	0.64940(19)	0.64387(18)	0.64972(15)	0.64367(15)
O3 *y*	0.9012(2)	0.90398(18)	0.90168(16)	0.90295(15)
Fe1 *z*	0.11001(3)	0.11129(3)	0.11005(3)	0.11103(3)
O2 *z*	0.44869(16)	0.45025(15)	0.44809(12)	0.44979(12)
O3 *z*	0.13541(14)	0.13469(13)	0.13541(10)	0.13426(11)
U_{11} Ca / Å^2	0.0084(5)	0.0146(6)	0.0093(4)	0.0135(4)
U_{11} Fe1 / Å^2	0.0101(3)	0.0168(3)	0.0086(2)	0.0158(2)
U_{11} Fe2 / Å^2	0.0105(4)	0.0240(5)	0.0098(3)	0.0231(4)
U_{iso} O1 / Å^2	0.0091(6)	0.0111(6)	0.0093(5)	0.0162(5)
U_{iso} O2 / Å^2	0.0108(4)	0.0196(5)	0.0099(3)	0.0193(4)
U_{iso} O3 / Å^2	0.0088(4)	0.0153(5)	0.0083(3)	0.0148(4)
U_{22} Ca / Å^2	0.0089(5)	0.0187(5)	0.0095(4)	0.0195(5)
U_{22} Fe1 / Å^2	0.0076(3)	0.0159(3)	0.0086(2)	0.0162(3)
U_{22} Fe2 / Å^2	0.0085(4)	0.0182(4)	0.0100(3)	0.0178(3)
U_{33} Ca / Å^2	0.0073(4)	0.0234(6)	0.0091(3)	0.0232(4)
U_{33} Fe1 / Å^2	0.0073(2)	0.0163(3)	0.00867(19)	0.0164(2)
U_{33} Fe2 / Å^2	0.0072(3)	0.0180(4)	0.0082(2)	0.0154(3)
U_{23} Fe1 / Å^2	0.0006(2)	0.0013(2)	0.00066(17)	0.00098(19)
U_{23} Fe2 / Å^2	0.0003(3)	0.0040(3)	0.0005(2)	0.0037(2)

Cmcm structural model as in Supplementary Table 2
